# Radiological unilateral pleuroparenchymal fibroelastosis as a notable late complication after lung cancer surgery: incidence and perioperative associated factors

**DOI:** 10.1093/icvts/ivac223

**Published:** 2022-08-22

**Authors:** Kenji Inafuku, Akimasa Sekine, Hiromasa Arai, Eri Hagiwara, Shigeru Komatsu, Tae Iwasawa, Toshihiro Misumi, Noritake Kikunishi, Michihiko Tajiri, Koji Okudela, Yasushi Rino, Takashi Ogura

**Affiliations:** Department of Thoracic Surgery, Kanagawa Cardiovascular and Respiratory Center, Yokohama, Japan; Department of Respiratory Medicine, Kanagawa Cardiovascular and Respiratory Center, Yokohama, Japan; Department of Thoracic Surgery, Kanagawa Cardiovascular and Respiratory Center, Yokohama, Japan; Department of Respiratory Medicine, Kanagawa Cardiovascular and Respiratory Center, Yokohama, Japan; Department of Respiratory Medicine, Kanagawa Cardiovascular and Respiratory Center, Yokohama, Japan; Department of Radiology, Kanagawa Cardiovascular and Respiratory Center, Yokohama, Japan; Department of Biostatistics, Yokohama City University School of Medicine, Yokohama, Japan; Department of Thoracic Surgery, Kanagawa Cardiovascular and Respiratory Center, Yokohama, Japan; Department of Thoracic Surgery, Kanagawa Cardiovascular and Respiratory Center, Yokohama, Japan; Department of pathology, Yokohama City University School of Medicine, Yokohama, Japan; Department of Surgery, Yokohama City University School of Medicine, Yokohama, Japan; Department of Respiratory Medicine, Kanagawa Cardiovascular and Respiratory Center, Yokohama, Japan

**Keywords:** Pleuroparenchymal fibroelastosis, Unilateral, Late complication, Surgery, Pleural effusion

## Abstract

**OBJECTIVES:**

Pleuroparenchymal fibroelastosis (PPFE) is a rare idiopathic interstitial pneumonia characterized by pleural-parenchymal involvement, predominantly in the upper lobes. Unilateral upper lung field pulmonary fibrosis (upper-PF) that is radiologically consistent with PPFE reportedly develops after lung cancer surgery in the operated side and presents many clinical characteristics in common with PPFE. However, the incidence and perioperative associated factors remain unclear.

**METHODS:**

All consecutive patients with lung cancer resected completely from 2008 to 2016 were investigated retrospectively. Pre-/postoperative characteristics were compared between patients with and without unilateral upper-PF. Cumulative incidence curves were estimated using competing risk analysis.

**RESULTS:**

Among the 587 included patients, 25 patients (4.3%) were diagnosed as unilateral upper-PF. The 3-, 5- and 10-year cumulative incidence of unilateral upper-PF was 2.3%, 3.3% and 5.3%, respectively. In multivariable analysis, male sex, presence of a pulmonary apical cap, lobar resection and low % vital capacity (%VC < 80%) were independent perioperative associated factors. The 10-year cumulative incidence was 6.3% in patients treated with lobar resection, 8.0% in male patients, 10.3% in patients with pulmonary apical cap and 14.5% in patients with low %VC. Postoperative pleural effusion at 6 months after surgery was much more common in the patients who later developed unilateral upper-PF (96.0% vs 24.2%). This pleural effusion persisted and was accompanied thereafter by pleural thickening and subpleural pulmonary fibrosis. During the clinical courses of 25 patients with unilateral upper-PF, 18 patients presented symptoms related to upper-PF and 6 patients died.

**CONCLUSIONS:**

Unilateral upper-PF is an occasional but under-recognized late complication after lung cancer surgery.

**Figure:**
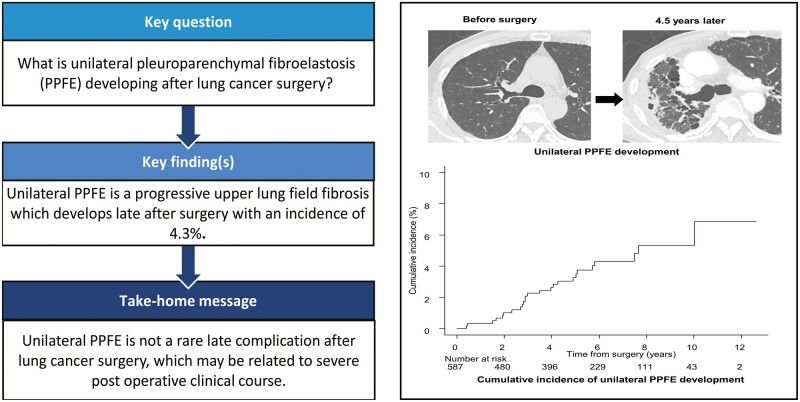

## INTRODUCTION

Pleuroparenchymal fibroelastosis (PPFE) is a rare form of interstitial lung disease characterized by pleural and subjacent parenchymal fibrosis predominantly in the bilateral upper lobes [1–3]. Patients with PPFE frequently have a low body mass index (BMI), deteriorated pulmonary function and poor prognosis [1, 3, 4]. In addition to idiopathic causes, a bone marrow, lung, or other organ transplant; radiotherapy; chemotherapy; recurrent lung infections; and an overactive immune system can cause PPFE [5].

More recently, upper lung field pulmonary fibrosis (upper-PF) radiologically consistent with PPFE has been reported to develop as a late complication after thoracic surgery [6, 7]. The upper-PF lesions were limited to the operated side but were similar to PPFE in terms of radiological and clinical characteristics. Most of the patients with unilateral upper-PF presented with respiratory symptoms and developed intra-/extrathoracic aberrant air suggestive of lung parenchymal air leak during their clinical courses [8], which might be related to the progression of unilateral upper-PF [7]. The prognosis was poor with a median survival time of 49.3 months, and all causes of death were respiratory diseases. Regarding respiratory complications after thoracic surgery, pneumonia is well known and can be caused by bacterial colonization of the atelectatic lung [9]. Pneumonia generally occurs in the early postoperative period and may improve with appropriate treatment. On the other hand, unilateral upper-PF occurs late after surgery [6, 7] and therefore may receive less attention compared to pneumonia. Although unilateral upper-PF is considered an important late complication after thoracic surgery, little is known about the incidence, mechanisms and perioperative-associated factors responsible for unilateral upper-PF development after lung cancer surgery. In addition, previous reports showed that almost all patients with unilateral upper-PF had a preoperative pulmonary apical cap in the apex of the lungs [6], and an autopsy showed findings consistent with postoperative chronic pleuritis [7]. However, the effect of a pulmonary apical cap and postoperative chronic pleuritis on disease development is also unclear. The purpose of this study was to determine the incidence, mechanisms and perioperative factors associated with the development of unilateral upper-PF.

## MATERIAL AND METHODS

### Ethics statement

The study protocol was approved by the ethics committee of Kanagawa Cardiovascular and Respiratory Center (KCRC-20–0052, approval date: 2021/02/24). The requirement for informed consent from individual patients was waived because it was a retrospective study.

### Study participants

All consecutive patients with lung cancer who underwent complete resection between January 2008 and December 2016 at Kanagawa Cardiovascular and Respiratory Center, Japan, were retrospectively investigated for eligibility. Complete resection was defined as demonstrating cancer-free surgical margins, both grossly and histologically. We excluded patients with a history of bilateral interstitial pneumonia; thoracic surgery, including cardiac, oesophageal and lung surgery; inflammatory lung disease, including mycobacteriosis and aspergillosis; and chemotherapy and chest radiotherapy for a previous malignancy. We also excluded patients who underwent pneumonectomy. For patients with lung cancer recurrence, we evaluated the clinical and radiological findings within the recurrence-free survival period, considering the effects of treatment for recurrent cancer. Patients with early recurrence within 6 months after surgery were also excluded. For patients with metachronous multiple lung cancer, we evaluated the clinical and radiological findings until just before the start of their treatment. Patients treated for multiple lung cancers within 6 months after surgery were also excluded.

Eligible patients were divided into 2 groups based on the development of unilateral upper-PF: the group with unilateral upper-PF and the group without unilateral upper-PF. Upper-PF was diagnosed when radiological findings obtained with computed tomography (CT) matched the criteria of “definite PPFE” or “consistent with PPFE” previously reported as follows: “Definite PPFE” was defined as demonstrating pleural thickening associated with subpleural fibrosis concentrated in the upper lobes, with markedly less or absent involvement of the lower lobes. “Consistent with PPFE” was defined as pleural thickening associated with subpleural fibrosis in the upper lobes, but the distribution of these changes was not concentrated in the upper lobes nor were there features of coexistent disease elsewhere [1]. These criteria were applied only for a unilateral lung in the operated side. The diagnosis of upper-PF was made based on the consensus of at least 2 board-certified chest surgeons, chest physicians and chest radiologists.

We then reviewed the medical records of all eligible patients and compared the clinical characteristics, including age, sex, smoking history, BMI, results of respiratory functional analysis, operative approach (video-assisted thoracoscopic surgery or open thoracotomy), operative procedure (lobar resection or sublobar resection), the use of a polyglycolic acid (PGA) sheet and fibrin glue, a preoperative pulmonary apical cap and adjuvant chemotherapy between the 2 groups.

### Operative procedure

Lobar resection is a lobectomy (removal of a single lobe) or bilobectomy (removal of 2 adjacent lobes). Sublobar resection is wedge resection or segmentectomy. We routinely used the PGA sheet (Neoveil large size; 10.0 x 10.0 cm: Gunze, Kyoto, Japan) and 3 ml or 5 ml of fibrin glue (Bolheal: Teijin, Tokyo, Japan or Beriplast P Combi-Set: CSL Behring, Tokyo, Japan) to cover the dissected hilum when we performed a lobectomy, a bilobectomy and a segmentectomy, regardless of a lung parenchymal air leak. In the case of wedge resection, we covered them to the point of the air leak only when we confirmed the presence of an air leak.

### Definition of pulmonary apical cap

A pulmonary apical cap is a wedge- and triangle-shaped opacity in the apex of the lung with broad pleural contact [10, 11]. The length of the pleural contact was reported to range from 7 mm to 6.0 cm on CT scans [10, 11]. Therefore, we evaluated the presence or absence of a preoperative pulmonary apical cap with a pleural contact of 5 mm or more on the operated side on CT.

### Radiological evaluation before and after lung cancer surgery

In all eligible patients, thin-sliced high-resolution CT (1 mm) was routinely performed before and after lung cancer surgery. During the postoperative follow-up period, chest radiography and CT scans were performed alternately every 3 months for the first 5 years; thereafter CT was performed every 6 months, typically for up to 10 years. All images were interpreted using Synapse (Fujifilm Medical Systems, Japan).

### Pathological evaluation of the background of the lung specimen in patients who developed unilateral upper-PF later

Histopathologic materials from all patients who developed unilateral upper-PF later were reviewed by a board-certified pathologist. The presence or absence of interstitial pneumonia in the area distant from the lung cancer was evaluated microscopically.

### Pleural effusion at 6 months after lung cancer surgery

A previous report of an autopsy of a patient with unilateral upper-PF showed chronic pleuritis and suggested that chronic pleuritis following thoracic surgery contributes to the development of unilateral upper-PF [7]. Because all eligible patients were routinely evaluated with CT every 6 months, we assessed the presence or absence of pleural effusion on CT at 6 months after surgery, regardless of the amount of pleural fluid.

### Statistical analyses

Descriptive statistics were expressed as n (%) or median and range. The Mann–Whitney U test and the Fisher exact test were used to compare continuous variables and categorical variables, respectively. A competing risk method was used to estimate the cumulative incidence of unilateral upper-PF. In this study, recurrence of lung cancer, treatment for metachronous multiple lung cancer or death of other diseases before unilateral upper-PF development was considered a competing risk event. Patients who had not developed unilateral upper-PF and had not died were censored at the time of the last follow-up. Gray’s test was used to test for differences in the cumulative incidence curves. The association between unilateral upper-PF and patient characteristics at lung cancer surgery were assessed using a proportional hazards model (Fine-Gray model). Statistical significance was set at a *P*-value < 0.05. Variables with *P*-values < 0.05 in univariable analysis were considered factors potentially associated with upper-PF development and used in subsequent multivariable analysis. Statistical analyses were conducted using EZR (Saitama Medical Center, Jichii Medical University, Saitama, Japan), which is a graphical user interface for R (The R Foundation for Statistical Computing, Vienna, Austria) [12].

## RESULTS

### Patient characteristics

Of the 943 patients with completely resected lung cancer, 587 patients were included. The study profile is shown in Supplementary Fig. 1. The median follow-up period was 5.1 years and the mean follow-up index was 0.84 (standard deviation: 0.23). Twenty-five patients (4.3%) developed unilateral upper-PF, and 562 (95.7%) did not. Patient characteristics are shown in Table 1. Twenty-five patients with unilateral upper-PF were significantly older (*P* = 0.035), more were men (*P* = 0.004) and more had a lower BMI (*P* = 0.014) than those without. Lobar resection was more frequently performed in patients with unilateral upper-PF than in those without (92.0% vs 73.1%, *p* = 0.036). The incidence of unilateral upper-PF development was 4.5% for lobectomy (19/426) and 50.0% for bilobectomy (4/8). The details of operative procedures for patients with unilateral upper-PF are shown in Table 2. Patients with unilateral upper-PF more commonly had a preoperative pulmonary apical cap than those without (84.0% vs 39.7%, *P* < 0.001). None of the patients received adjuvant radiotherapy.

**Table 1:** Patient characteristics

Characteristics	All cases	With unilateral upper-PF	Without unilateral upper-PF	*P*-value
	(n = 587)	(n = 25)	(n = 562)	
Age (years)				
Median (range)	69 (25–86)	73 (55–85)	69 (25–86)	0.035
< 70, n (%)	307 (52.2)	8 (32.0)	299 (53.2)	0.042
≥ 70	280 (47.7)	17 (68.0)	263 (46.8)	
Sex, n (%)				
Male	331 (56.3)	21 (84.0)	310 (55.1)	0.004
Female	256 (43.6)	4 (16.0)	252 (44.8)	
Smoking history, n (%)				
Yes	356 (60.6)	19 (76.0)	337 (60.0)	0.143
No	231 (39.4)	6 (24.0)	225 (40.0)	
BMI (kg/m^2^)				
Median (range)	22.2 (15.2–43.9)	20.3 (16.0–26.1)	22.3 (15.2–43.9)	0.014
%VC (%)				
Median (range)	104.9 (51.3–169.8)	100.7 (72.4–129.5)	105.1 (51.3–169.8)	0.056
≥ 80, n (%)	561 (95.6)	22 (88.0)	539 (95.9)	0.093
< 80	26 (4.4)	3 (12.0)	23 (4.1)	
FEV_1.0_% (%)				
Median (range)	73.1 (29.0–100.0)	74.1 (45.6–99.4)	73.0 (29.0–100.0)	0.325
≥ 70, n (%)	376 (64.1)	15 (60.0)	361 (64.2)	0.674
< 70	211 (35.9)	10 (40.0)	201 (65.8)	
Operative approach, n (%)				
VATS	554 (94.3)	23 (92.0)	531 (94.5)	0.645
Open thoracotomy	33 (5.6)	2 (8.0)	31 (5.5)	
Operative procedure, n (%)				
Lobar resection	434 (73.9)	23 (92.0)	411 (73.1)	0.036
Sublobar resection	153 (26.0)	2 (8.0)	151 (26.9)	
Use of PGA sheet and fibrin glue				
Yes	542 (92.3)	25 (100)	517 (92.0)	0.246
No	45 (7.7)	0 (0)	45 (8.0)	
Pulmonary apical cap, n (%)				
Absent	343 (58.4)	4 (16.0)	339 (60.3)	< 0.001
Present	244 (41.6)	21 (84.0)	223 (39.7)	
Adjuvant chemotherapy				
Yes	47 (80.0)	1^*^ (4.0)	46 (8.2)	0.712
No	540 (20.0)	24 (96.0)	516 (91.8)	

* Uracil-tegafur was administered orally.

BMI: body mass index; FEV_1.0_%: % forced expiratory volume in 1 s; lobar resection: lobectomy or bilobectomy; %VC: % vital capacity; PGA: polyglycolic acid; sublobar resection: wedge resection or segmentectomy; upper-PF: upper lung field pulmonary fibrosis; VATS: video assisted thoracoscopic surgery.

**Table 2:** Operative procedures of the patient with unilateral upper-PF

Operative procedures	n
Lobar resection	23
Lobectomy	19
Right upper lobectomy	4
Right middle lobectomy	1
Right lower lobectomy	8
Left upper lobectomy	5
Left lower lobectomy	1
Bilobectomy	4
Right middle and lower lobectomy	2
Right upper and middle lobectomy	2
Sublobar resection	2
Left upper division segmentectomy	1
Left lower lobe wedge resection	1

upper-PF: upper lung field pulmonary fibrosis.

### Cumulative incidence of unilateral upper-PF after lung cancer surgery

**Figure 1:**
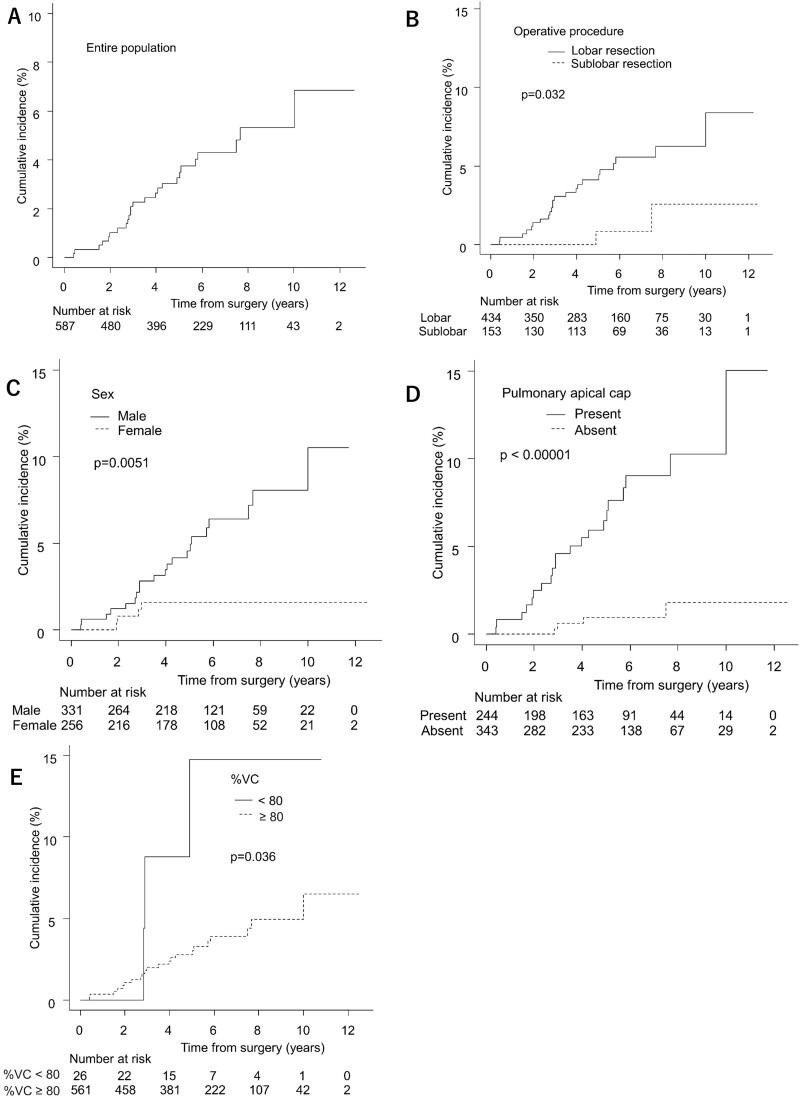
(**A–E**) Cumulative incidence curves of unilateral upper-PF after lung cancer surgery. upper-PF: upper lung field pulmonary fibrosis; VC: vital capacity.

The cumulative incidence of unilateral upper-PF gradually increased 2 years after lung cancer surgery (Fig. 1A). For the entire population, the 3-, 5-, 10- and 12-year cumulative incidences of unilateral upper-PF were 2.3%, 3.3%, 5.3% and 6.9%, respectively.

### Pleural effusion at 6 months after lung cancer surgery

We investigated the presence or absence of postoperative pleural effusions at 6 months after surgery and found that pleural effusion was much more common in patients with unilateral upper-PF than in those without (96.0% vs 24.2%, *P* < 0.001) (Table 3). This trend was also observed regardless of the operative procedure (lobar resection and sublobar resection). Of note, pleural effusion in all patients with unilateral upper-PF persisted and was accompanied by pleural thickening and fibrosis adjacent to the pleura.

**Table 3:** Postoperative pleural effusion 6 months after surgery

Pleural effusion	With unilateral upper-PF	Without unilateral upper-PF	*P*-value
All lung cancer surgery (n = 587)			
Present	24 (96.0)	136 (24.2)	< 0.001
Absent	1 (4.0)	426 (75.8)	
Lobar resection (n = 434)			
Present	22 (95.7)	111 (27.0)	< 0.001
Absent	1 (4.3)	300 (73.0)	
Sublobar resection (n = 153)			
Present	2 (100)	8 (5.3)	0.0039
Absent	0 (0)	143 (94.7)	

lobar resection: lobectomy or bilobectomy; sublobar resection: wedge resection or segmentectomy; upper-PF: upper lung field pulmonary fibrosis.

### Clinical and radiological courses of patients with unilateral upper-PF

The median interval from lung cancer surgery to the diagnosis of unilateral upper-PF was 36.3 months (range, 4.8–121.8) (Table 4). We carefully examined the radiological findings on CT scans in order to detect intra-/extrathoracic aberrant air such as subcostal and pneumothorax air. Aberrant air was detected in 17 patients (68.0%), although the degree of all aberrant air was slight, as shown in Supplementary Fig. 2. During their radiological courses, the upper-PF lesions initially showed only slight fibrosis adjacent to the pleura in all patients. However, the upper-PF lesions deteriorated with cystic changes in 20 patients (80%), which occasionally resulted in pulmonary aspergillus and nontuberculous mycobacterium infection, whereas 5 patients (20%) presented with radiological deterioration without any cystic changes. Eighteen patients (72.0%) had some adverse events caused by unilateral upper-PF. The typical case of pulmonary aspergillus infection following unilateral upper-PF progression is shown in Supplementary Fig. 3. The median follow-up period after unilateral upper-PF diagnosis was 29.7 months (range, 3.1–57.6). During this period, 6 patients (24.0%) died of unilateral upper-PF-related causes.

**Table 4:** Clinical and radiological courses of 25 patients with unilateral upper-PF

Median interval time from surgery to diagnosis of upper-PF (range)	36.3 months
	(4.8–121.8)
Aberrant air, n (%)	
Present	17 (68.0)
Absent	8 (32.0)
Radiological findings of upper-PF, n (%)	
Cystic change	20 (80.0)
Only fibrosis lesion	5 (20.0)
Subsequent complication related to upper-PF, n (%)	
Present	18 (72.0)
Complications (a cumulative total of)	
Progressive respiratory distress	9
Progressive body weight loss	9
Pneumonia	6
Pulmonary aspergillus	4
Nontuberculous mycobacterium infection	1
Absent	5 (20.0)
Unknown	2 (8.0)
Unilateral thoracic deformity, n (%)	
Present	21 (84.0)
Absent	4 (16.0)
Prognosis, n (%)	
Alive	19 (76.0)
Dead	6 (24.0)
Causes of death	
Pulmonary aspergillus	2
Hypercapnic chronic respiratory failure	2
Pneumonia	2

upper-PF: upper lung field pulmonary fibrosis.

### The pathological background of lung specimens resected for lung cancer in 25 patients who later developed unilateral upper-PF

Interstitial pneumonia was pathologically observed in 2 patients (8.0%) despite no radiological evidence of interstitial shadow. One was the usual interstitial pneumonia and the other was indeterminate for interstitial pneumonia. In addition, centrilobular emphysematous change was commonly observed in 18 patients (72%).

### Perioperative factors associated with the development of unilateral upper-PF

Multivariable analysis showed that male sex (subdistribution hazard ratio [SHR] = 4.25, *P* = 0.010), %VC < 80% (SHR = 3.56, *P* = 0.047), lobar resection (SHR = 4.31, *P* = 0.038) and presence of pulmonary apical cap (SHR = 6.47, *P* = 0.0032) were independent perioperative associated factors for unilateral upper-PF development (Table 5). A proportional hazards model (Fine-Gray model) could not be performed concerning the use of a PGA sheet and fibrin glue because no patients developed upper-PF without the use of such materials. On the other hand, Gray’s test (*P* = 0.13) showed no significant difference in the cumulative incidence of upper-PF development between patients with and without the use of such materials.

**Table 5:** Univariable and multivariable analyses for perioperative associated factors for unilateral upper-PF

Variables	Univariable analysis			Multivariable analysis		
	SHR	95% CI	*P*-value	SHR	95% CI	*P*-value
Age (≥ 70)	2.50	1.09–5.76	0.031	2.07	0.845–5.02	0.11
Sex (male)	4.11	1.41–12.00	0.0096	4.25	1.40–12.84	0.010
Smoking history (yes)	2.13	0.85–5.32	0.11			
BMI^*^	0.85	0.75–0.96	0.0078	0.89	0.76–1.05	0.18
%VC (< 80%)	3.39	1.02–11.26	0.047	3.56	1.02–2.46	0.047
FEV_1.0_% (< 70%)	1.25	0.33–2.79	0.57			
Operative approach (open thoracotomy)	1.37	0.33–5.65	0.67			
Operative procedure (lobar resection)	4.20	1.01–17.58	0.049	4.31	1.08–17.15	0.038
Use of PGA sheet and fibrin glue	N/E					
Pulmonary apical cap (presence)	7.71	2.64–22.52	0.00019	6.47	1.87–22.30	0.0032
Adjuvant chemotherapy (yes)	0.64	0.08-4.81	0.66			

^*^ upper-PF: upper lung field pulmonary fibrosis; BMI: body mass index; CI: confidence interval; FEV_1.0_%: % forced expiratory volume in 1 s; lobar resection: lobectomy or bilobectomy; N/E: not evaluable because no patients developed upper lung field pulmonary fibrosis without the use of PGA sheet and fibrin glue; %VC: % vital capacity; PGA: polyglycolic acid; SHR: subdistribution hazard ratio.

The cumulative incidence curves and the details of the cumulative incidence according to perioperative associated factors are shown in Fig. 1B-E and Table 6. The 10-year cumulative incidence was 6.3% in patients treated with lobar resection, 8.0% in male patients, 10.3% in patients with pulmonary apical cap and 14.5% in patients with low %VC, respectively.

**Table 6:** Cumulative incidence of unilateral upper-PF according to perioperative characteristics

	Cumulative incidence (95% C.I.)		
Perioperative characteristics	3-year	5-year	10-year
Entire population	2.3% (1.3–3.8)	3.3% (2.0–5.0)	5.3% (3.4–7.9)
Operative procedure			
Lobar resection	3.1% (1.7–5.1)	4.1% (2.5–6.3)	6.3% (3.9–.4)
Sublobar resection	0.0% (0.0–0.0)	0.8% (0.1–4.2)	2.6% (0.4–8.7)
Sex			
Male	2.8% (1.4–5.1)	4.5% (2.6–7.3)	8.0% (4.9–12.2)
Female	1.6% (0.5–3.8)	1.6% (0.5–3.8)	1.6% (0.5–3.8)
Apical cap^*^			
Present	4.6% (2.4–7.8)	6.5% (3.8–10.2)	10.3% (6.3–15.4)
Absent	0.6% (0.1–2.1)	1.0% (0.3–2.6)	1.8% (0.5–4.7)
%VC			
< 80%	8.8% (1.4–25.0)	14.5% (3.3–33.5)	14.5% (3.3–33.5)
≥ 80%	2.0% (1.1–3.5%)	2.8% (1.6–4.5%)	4.9% (3–7.5)

* On the operated side.

CI: confidence interval; lobar resection: lobectomy or bilobectomy; sublobar resection: wedge resection or segmentectomy; upper-PF: upper lung field pulmonary fibrosis; VC: vital capacity.

### Radiological courses of 2 patients with unilateral upper-PF

**Figure 2:**
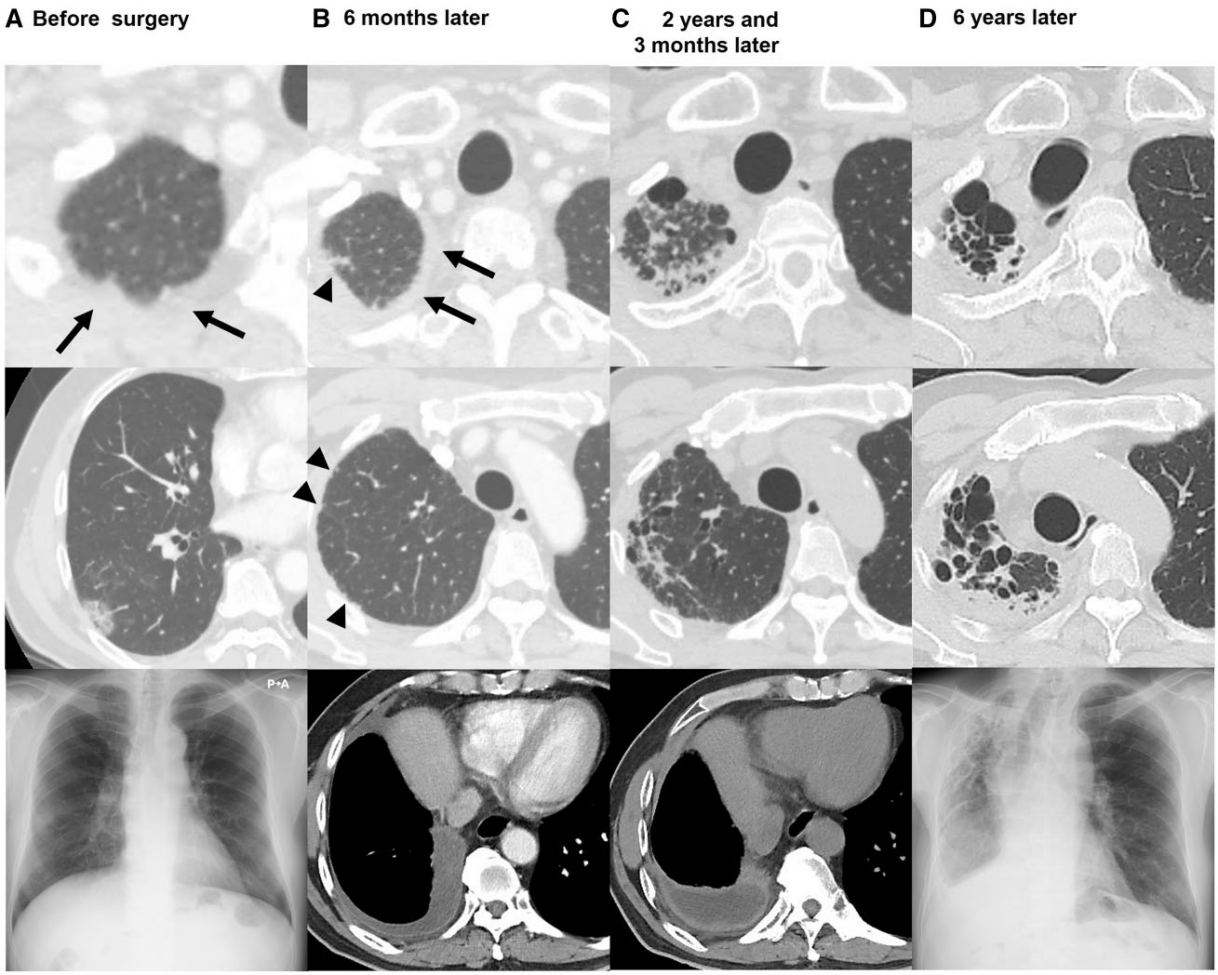
Radiological course of a 68-year-old male patient who underwent right lower lobectomy for stage IA adenocarcinoma. (**A**) Before surgery, computed tomography showed a pulmonary apical cap (arrow) and a mixed ground-grass opacity in the right lower lobe. A chest radiograph showed a ground-grass opacity in the right middle field of the lung. (**B**) Postoperative computed tomography 6 months after surgery demonstrated involvement of the pleura (arrow) and subpleural parenchyma (arrowhead) in the right upper lobe and right pleural effusion. (**C**) At 2 years and 3 months after surgery, a subpleural parenchymal lesion in the right upper lobe appeared to be deteriorated with cystic change and thickened pleural effusion. (**D**) At 6 years after surgery, the cystic lesion in the right upper lung field was obviously deteriorated with unilateral thoracic deformity.

Figure 2 shows the radiological course of a 68-year-old male who underwent right lower lobectomy for stage IA lung adenocarcinoma. The patient was diagnosed with unilateral upper-PF 2 years and 3 months after the initial surgery. The cystic lesion deteriorated and resulted in nontuberculous mycobacteria infection and progressive body weight loss of 17 kg in 3 years.

**Figure 3:**
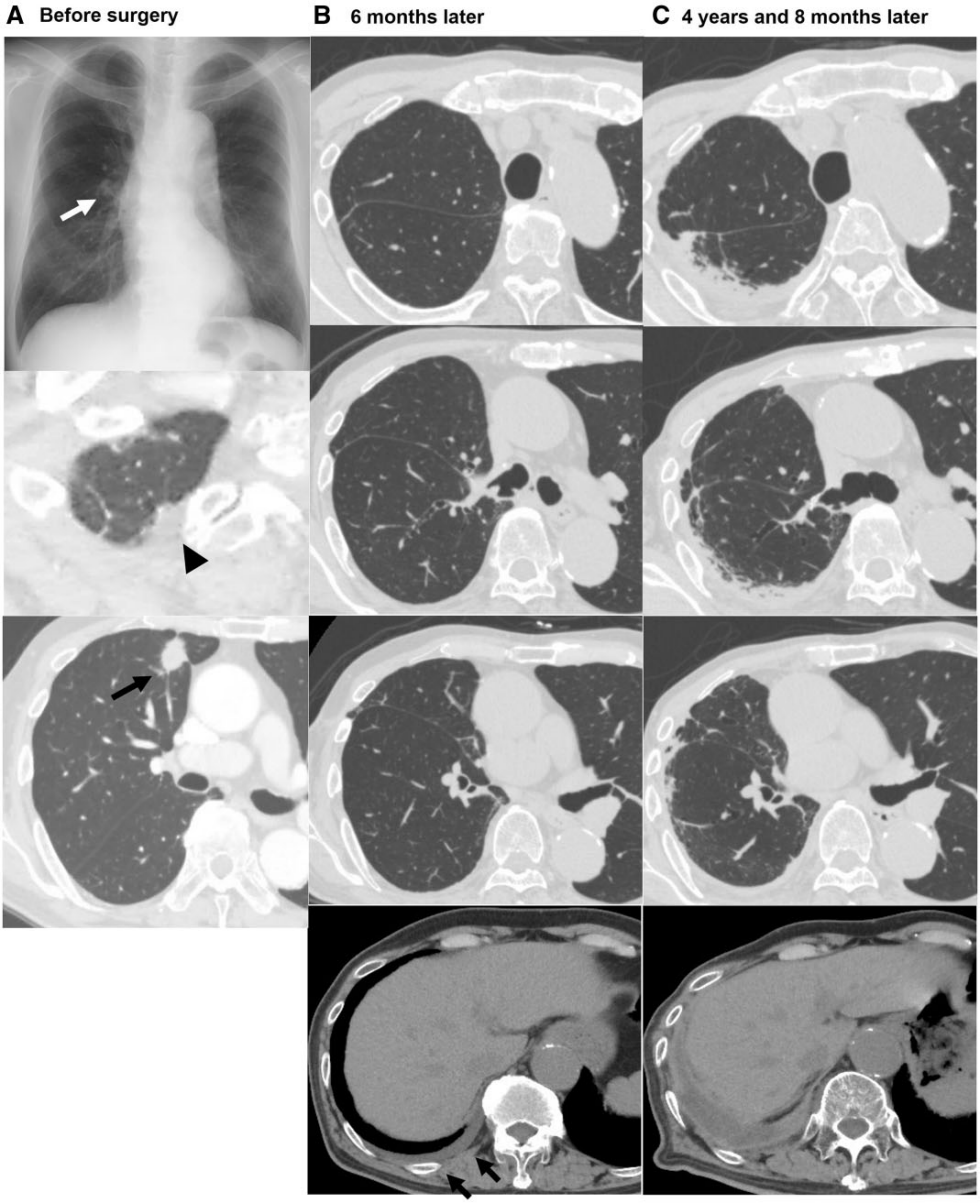
Radiological course of a 78-year-old male patient who underwent a right upper lobectomy for stage IB adenocarcinoma. (**A**) Preoperative radiological radiograph showed a small nodule (white arrow) in the right middle lung field, and chest computed tomography demonstrated a pulmonary apical cap (arrowhead) and a nodule (black arrow) in the right upper lobe. (**B**) Postoperative computed tomography at 6 months after surgery showed no abnormal shadows except for a small amount of pleural effusion (arrow) on the operated side. (**C**) At 4 years and 8 months after surgery, pleural thickening and subpleural fibrosis lesion emerged in the right upper lung field with unilateral thoracic deformity and thickened pleural effusion.

Figure 3 shows the radiological course of a 78-year-old male who underwent right upper lobectomy for stage IB lung adenocarcinoma. The unilateral upper-PF lesion apparently worsened without cystic change, and the patient was finally diagnosed with unilateral upper-PF. Although he was asymptomatic during the radiological course, a unilateral thoracic deformity apparently emerged.

## DISCUSSION

The present study identified 4 clinical findings. First, the incidence of unilateral upper-PF development was 4.3% among all patients having lung cancer surgery, 4.5% for lobectomy and 50.0% for bilobectomy. The cumulative incidence gradually increased after lung cancer surgery, and the 10-year cumulative incidence was 5.3%. Second, 72% of the patients with unilateral upper-PF had some adverse events associated with the lesion. Third, pleural effusion at 6 months postoperatively was much more common in patients who later developed unilateral upper-PF than in those who did not. Fourth, multivariable analysis revealed male sex, low %VC, lobar resection and the presence of a pulmonary apical cap as perioperative factors associated with the development of unilateral upper-PF. These results provide the following 3 characteristics associated with unilateral upper-PF:

First, unilateral upper-PF is an occasional but under-recognized late complication after lung cancer surgery. Although there are many studies on early complications such as pneumonia after lung cancer surgery [9, 13, 14], reports on late complications are limited. The present study showed that the 10-year cumulative incidence was 6.3% in patients treated with lobar resection, 8.0% in male patients, 10.3% in patients with pulmonary apical cap and 14.5% in patients with low %VC. Further, the unilateral upper-PF usually resulted in a severe clinical course, including infection and respiratory failure. Therefore, unilateral upper-PF is a notable late complication after lung cancer surgery. There is a report on “unilateral fibrobullous change” as a late complication of open lobectomy for lung cancer [15]. This complication developed at the apex of the re-expanded residual lobes an average of 2 years after the lobectomy with a prevalence of 3.0%, although these authors did not perform a competing risk method. “Unilateral fibrobullous change” seems to have some characteristics similar to those of unilateral upper-PF. However, careful evaluation of radiological changes by thin-sliced CT findings every 6 months indicated that the upper-PF lesions initially showed only slight fibrosis adjacent to the pleura in all patients, and gradually became aggravated with or without cystic change. Thus, fibrobullous change may be one of the manifestations of unilateral upper-PF in the advanced stage.

Second, chronic pleuritis may be the first step in the unilateral development of upper-PF. To date, the mechanisms underlying unilateral upper-PF development after lung cancer surgery remain unclear. The autopsied case of unilateral upper-PF showed evidence of chronic pleuritis as well as secondary PPFE [7]. In addition, the previous study showed that almost all patients with unilateral upper-PF had aberrant air emergence, suggestive of lung parenchymal air leak on CT during their clinical course [7]. Therefore, some visceral pleural disorders may contribute to the progression of unilateral upper-PF. In the present study, almost all patients who later developed unilateral upper-PF had had pleural effusions in the residual space 6 months after surgery. The pleural effusion persisted, accompanied by pleural thickening and subpleural pulmonary fibrosis. These results indicate that chronic pleuritis contributes to the development of unilateral upper-PF. In fact, there was a case of bilateral tuberculous pleuritis progressing to upper-lobe-predominant pulmonary fibrosis mimicking PPFE 3 years thereafter [16], which supports our hypothesis. In addition, a PGA sheet and fibrin glue were used in all patients who later developed upper-PF. Because fibrin glue reportedly induced eosinophilic pleural effusion after lung resection [17], a PGA sheet or fibrin glue may play an important causal role in the development of upper-PF although the difference was not significant by Gray’s test (p=0.13).

Third, chest physicians and surgeons should be aware that the development of unilateral upper-PF may be correlated with perioperative patient characteristics including male sex, pulmonary apical cap, low %VC and lobar resection. To our knowledge, there is no definite reason for the predominance of male sex in the development of this disease. However, almost all patients with the above-mentioned “unilateral fibrobullous change” were male [15], which indicates that male sex is associated with disease development. With regard to low %VC and a preoperative pulmonary apical cap, patients with PPFE commonly have low %VC [1, 3, 4, 18], and a pulmonary apical cap has the same histological characteristics as PPFE despite being an anatomically localized, non-progressive lesion [1, 10, 19]. In addition, a pulmonary apical cap has been reported to be potentially caused by ischaemia in the upper lobes and low-grade inflammation in the lung parenchyma [1, 18] and is considered a potential risk factor for PPFE [20]. Therefore, we suppose that patients with low %VC and a pulmonary apical cap have some potential pathophysiological factors in common with those with PPFE. Furthermore, our results showed that lobar resection was also associated with unilateral upper-PF development. Generally, lobectomy, especially bilobectomy, is a wide resection of the lung compared to other procedures and is correlated with postoperative residual space. This postoperative residual space may cause persistent pleural effusion, which results in chronic pleuritis [21].

The limitations of this study include the following: a single-centre retrospective study, a small number of patients with unilateral upper-PF, a lack of pathological evaluations of the unilateral upper-PF lesions and no autopsy evaluations of the 6 patients who died. In addition, because no patients developed upper-PF without the use of a PGA sheet and fibrin glue, a proportional hazards model could not be used. Further study is needed to elucidate more detailed clinical and radiological characteristics, mechanisms and perioperative risk factors of unilateral upper-PF.

## CONCLUSION

Unilateral upper-PF is an occasional but under-recognized late complication after lung cancer surgery. This complication may be correlated with perioperative patient characteristics including male sex, low %VC, pulmonary apical cap and lobar resection.

## Supplementary Material

ivac223_Supplementary_Data

## Data Availability

All relevant data are within the manuscript and its supporting information files.
