# Supplementary material for: Radiological unilateral pleuroparenchymal fibroelastosis as a notable late complication after lung cancer surgery: incidence and perioperative associated factors
Source: Interact Cardiovasc Thorac Surg. 2022 Aug 22;35(4):ivac223. doi: 10.1093/icvts/ivac223 (PMC9487195; doi:10.1093/icvts/ivac223)
Supplement: ivac223_Supplementary_Data [file ivac223_supplementary_data.docx]

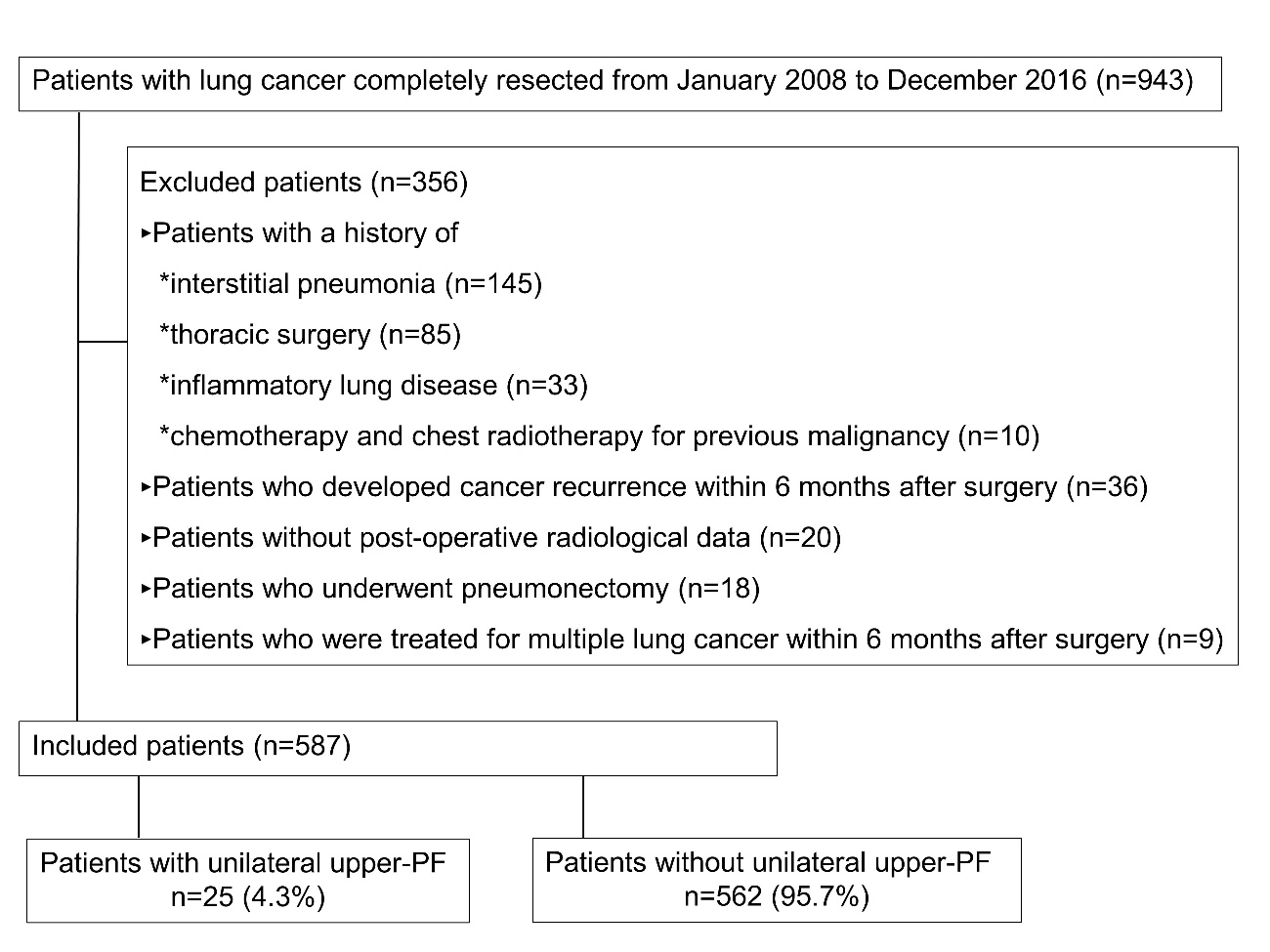


Supplementary Figure 1. Patient flow

upper-PF: upper lung field pulmonary fibrosis


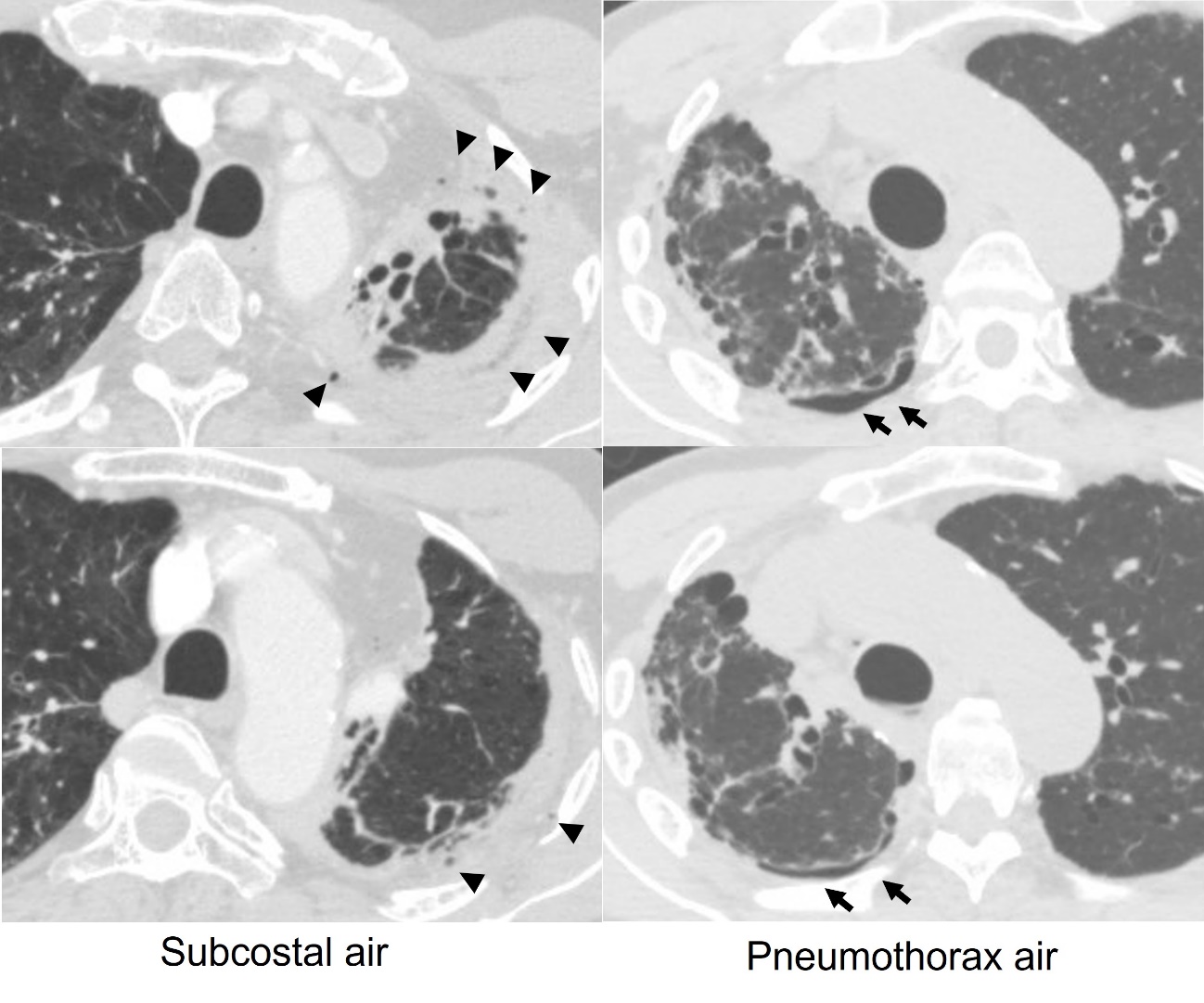


Supplementary Figure 2. Aberrant air


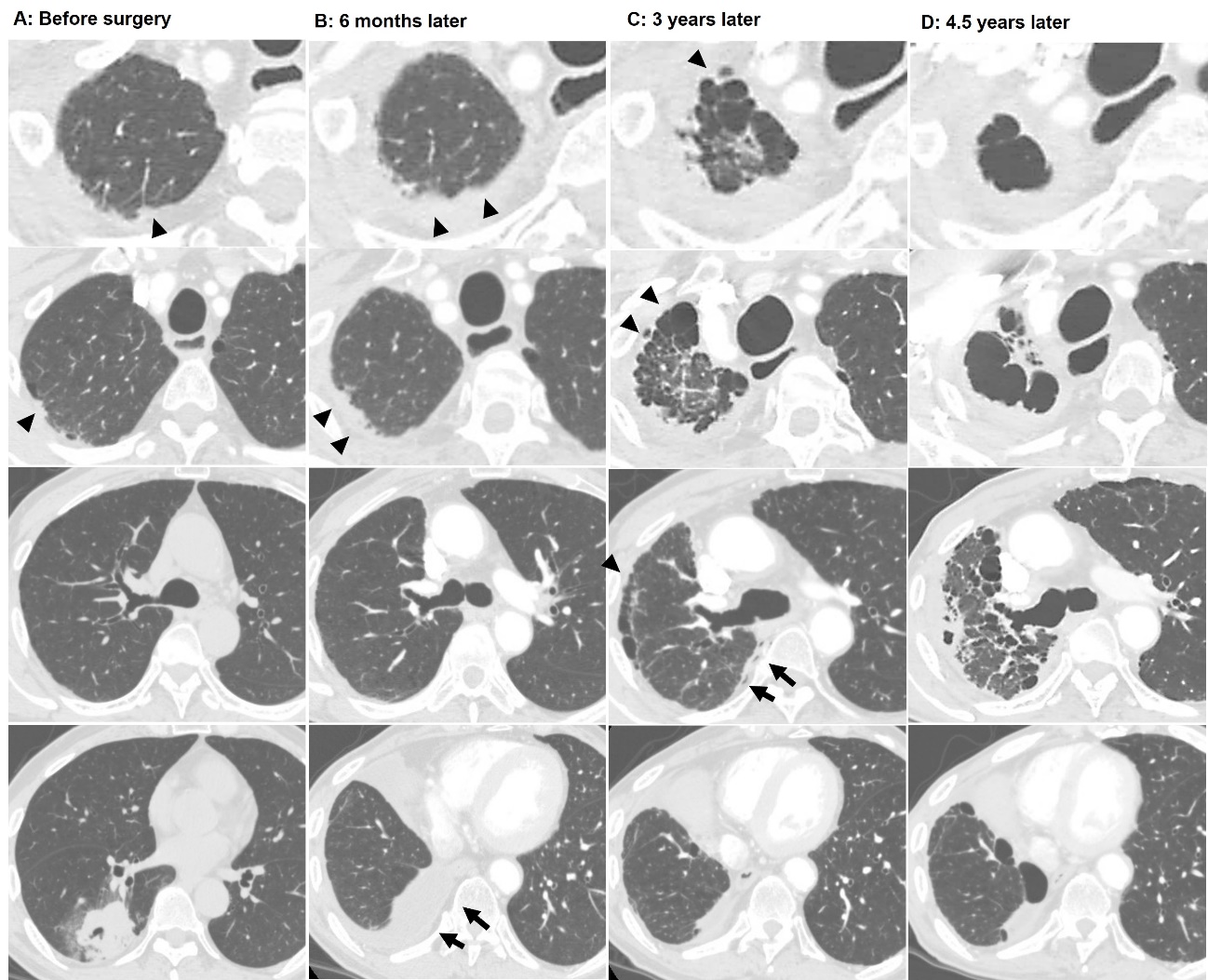


Supplementary Figure 3. A radiological course of a 78-year-old male who underwent right upper lobectomy for stage IB lung adenocarcinoma.

A: Preoperative chest computed tomography (CT) showed a mass in the right lower lobe a pulmonary apical cap (arrowhead) in the right upper lobe.

B: Chest CT at 6 months after surgery demonstrate a slight involvement of the pleura and subpleural parenchyma (arrowhead) in the right upper lobe and right pleural effusion (arrow).

C: At 3 years after surgery, chest CT showed subpleural cystic change (arrowhead), aberrant subcostal air (arrow) and unilateral thoracic deformity with thickened pleural effusion.

D: At 4.5 years after surgery, the cystic lesion was obviously deteriorated and infected with *Aspergillus fumigatus*.
